# A Randomized Clinical Trial of Levonorgestrel Intrauterine System with or without Metformin for Treatment of Endometrial Hyperplasia without Atypia in Indian Women

**DOI:** 10.31557/APJCP.2021.22.3.983

**Published:** 2021-03

**Authors:** Ramya Dinnekere Ravi, Jasvinder Kalra, Radhika Srinivasan, Rashmi Bagga, Vanita Jain, Vanita Suri, Naresh Sachdeva

**Affiliations:** 1 *Department of Obstetrics & Gynaecology, Post Graduate Institute of Medical Education and Research, Chandigarh, India. *; 2 *Department of Cytology & Gynecological Pathology, Post Graduate Institute of Medical Education and Research, Chandigarh, India. *; 3 *Department of Endocrinology, Post Graduate Institute of Medical Education and Research, Chandigarh, India. *

**Keywords:** Endometrial hyperplasia, metformin, levonorgestrel, LNG-IUS, endometrial hyperplasia without atypia

## Abstract

**Background:**

Endometrial cancer is the second most frequent genital malignancy in women, which is showing a constant rise all over world. Endometrial hyperplasia is the precursor of endometrial cancer. Levonorgestrel intrauterine system is the first line management in patients with endometrial hyperplasia without atypia. Metformin has shown to reverse endometrial hyperplasia, but its effectiveness and safety in endometrial hyperplasia is uncertain.

**Objective:**

To compare the efficacy in terms of histopathological response, clinical response and safety at the end of 6 months in patients with endometrial hyperplasia without atypia managed with levonorgestrel intrauterine system alone versus patients managed with levonorgestrel intrauterine system plus metformin.

**Methods:**

The randomized control trial was conducted on 51 cases of endometrial hyperplasia without atypia. Twenty-five subjects were prescribed metformin 500mg twice daily with levonorgestrel intrauterine system and 26 subjects, with levonorgestrel intrauterine system only for 6 months. At the end of 6 months, endometrial sampling was performed for histopathological response.

**Results:**

Clinical response was observed in 23 of 25 subjects in metformin group and 22 of 24 in levonorgestrel only group. The metformin group responded significantly with amenorrhea (p= 0.0053), while levonorgestrel only group responded with regular cycles (p=0.027). At the end of study, of 46 subjects available for histopathological evaluation, 100% subjects in metformin group and 95.45% in levonorgestrel only group (p=0.47826) showed complete response. The metformin group had a significant reduction in body mass index at end of study [P = 0∙023, 95% confidence interval (-1.7802, -0.1418)].

**Conclusion:**

No significant difference in regression of endometrial hyperplasia was observed on adjunctive use of metformin but a significant reduction in BMI was observed. Use of metformin in obese patients may improve the treatment response.

## Introduction

Endometrial Hyperplasia (EH) refers to a spectrum of morphological and biological alterations of endometrial glands and stroma, with exaggerated physiological state at one end of the spectrum and carcinoma in-situ on the other end. The most common presentation of EH is Abnormal Uterine Bleeding (AUB), usually sufficient to interfere with the quality of life. The World Health Organization (WHO) 2014 classification for EH has divided EH into EH without atypia and EH with atypia (Carcangiu et al., 2014). EH without atypia is a benign change with no underlying genetic alterations and it reverts back to normal after the endocrine milieu has normalized. It is clinically important as the risk of progression to Endometrial Cancer (EC) is 1-3% (Kurman et al., 1985). The key goal of management of EH is reversion to normal endometrium and prevention of development of carcinoma. The available management options include observation only with follow up or medical management like progestins, gonadotropin-releasing hormone analogues, metformin, ovulation induction and surgery (Chandra et al., 2016).

Management of EH has evolved over years. Simple EH without atypia was initially treated with surveillance only. Following the demonstration of risk of progression to cancer, newer modalities of treatment were introduced. The management evolved from cyclical to continuous oral progestins and currently the first line of management is Levonorgestrel intrauterine system (LNG-IUS) as recommended by Royal College of Obstetricians and Gynaecologists (RCOG) (Royal College of Obstetricians and Gynaecologists [RCOG], 2016).

Insulin resistance is a well-studied aetiopathological feature associated with EH, which can be targeted for the management (Kaya et al., 2019). Metformin is used widely in management of insulin resistance and it’s anti-proliferative, anti-invasive, anti-metastatic and anti-estrogenic effect on the endometrium makes it a logical treatment option (Tan et al., 2011; Cantrell et al., 2010). The property of metformin to induce progesterone receptors in endometrium may also help to overcome the progestin resistance (Xie et al., 2011). Various in vitro studies have shown that metformin causes cell cycle arrest and inhibits oestrogen dependent proliferation (Zhang et al., 2009). In recent years following the case report of use of metformin to treat EH (Session et al., 2003), various trials have come up with metformin as a management option for EH. Recent studies by Sharifzadeh et al. and Tabrizi et al. have shown that metformin can be used as an effective alternate management option for treatment of EH (Sharifzadeh et al., 2017; Tabrizi et al., 2014). 

There was a need to study the effect of adding metformin to the standard treatment of EH without atypia in a prospective randomized trial. The current study intends to compare the efficacy of LNG-IUS plus metformin versus LNG-IUS alone in terms of histopathological response, clinical response and the adverse effects in patients with EH without atypia. 

## Materials and Methods

This is a randomized prospective interventional study conducted in the Department of Obstetrics and Gynaecology and the Department of Pathology, Post Graduate Institute of Medical Education and Research, Chandigarh, India, from July 2016 to June 2018. Approval from the institutional ethics committee was obtained and the details have been uploaded in the clinical trial registry of India (CTRI/2017/05/008548).

Patients who presented with AUB and had a histopathological diagnosis of EH without atypia (Carcangiu et al., 2014) after Endometrial Biopsy (EB) were approached. Patients who understood the study design and were able to comply with the EB at specified interval were included in the study after obtaining an informed written consent. Patients who were 1) on progestin therapy for more than 15 days, 2) with uterine or extra uterine malignancy 3) with renal dysfunction, liver disease or diabetes mellitus 4)with history of hypersensitivity to metformin or discontinuation due to adverse effects 5) pregnancy or lactation, 6) recent (< 4 weeks) or active documented Pelvic Inflammatory Disease (PID) or cervical infection 7) with immune-suppression 8) abnormal Pap smear 9) contraindication to metformin or progestin or IUS were excluded from the study.

*Methodology*

Complete medical history, findings of general physical examination, systemic and gynaecological examination were recorded on a pre-designed proforma. Body Mass Index (BMI) of the patients was calculated. Patient’s haematological and biochemical parameters such as hemogram, coagulogram, fasting and postprandial blood sugars, fasting insulin level, thyroid profile, renal function test, liver function test and lipid profile were noted for any abnormalities. Insulin resistance using fasting blood sugar and fasting insulin level was calculated using the HOMA model [HOMA1-IR = (fasting plasma insulin * fasting plasma glucose)/22∙5] (Matthews et al., 1985). Fifty-one recruited patients were randomized by asking them to pick up an envelope from a set of similar looking pre-sealed opaque envelopes prepared by a third party into LNG-IUS with metformin group (Group M) and LNG-IUS alone group (Group L). Patients in both the groups underwent LNG-IUS (MIRENA, manufactured by Bayer Oy, Finland and imported and marketed by Bayer Zydus Pharma Pvt. Ltd. which contains 52mg Levonorgestrel with a release rate of approximately 20µg per day) insertion under aseptic precautions. The patients in group M were prescribed tablet metformin 500mg once a day for 1 week followed by twice a day for the rest of the duration of the study in addition to LNG-IUS. A pictorial menstrual diary was provided to all the patients to document the menstrual bleeding patterns for 6 months. 

Treatment response was assessed by repeat EB after six months of treatment. During the response assessment by histopathological examination, the pathologists were unaware of the management that the patient had received. Lipid profile, insulin resistance, hemogram, coagulogram, liver and renal function tests and BMI were reassessed to evaluate and compare the adverse effects among the groups. 

The primary outcome was response to treatment compared between the groups as complete response, no response and progressive disease. A complete response was defined as reversion of EH to proliferative or secretory endometrium, no response as the persistence of EH, and progressive disease as the appearance of atypia or EC. In secondary outcome, the comparison was made between the two groups regarding regression of menstrual symptoms (clinical response), effect on BMI and other biochemical parameters, need for the requirement of additional oral progesterone, adverse effects and patients opting for hysterectomy during the treatment period. 

The distribution of the variables was tested with the Shapiro-Wilk test/Kolmogorov-Smirnov tests of normality. Group comparisons of values of skewed data were made with the Mann Whitney test for the two groups. Independent t-test was applied for comparison of the two groups when data was normally distributed. Group comparisons were made with the Chi-Square test or Fisher’s exact test. Change was calculated for the variables to see change by the formula (post - pre). A P value < 0∙05 was considered signiﬁcant. Analysis was conducted using IBM SPSS STATISTICS (version 22.0).

## Results

**Figure 1:**
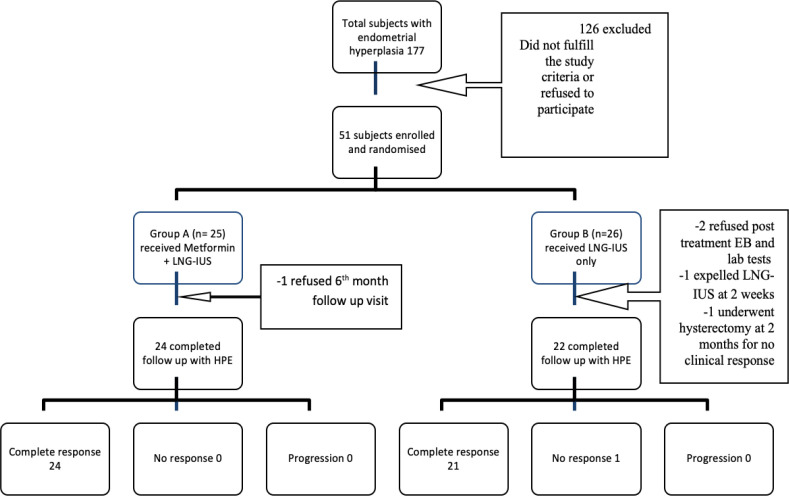
Study Design Flow Chart

During the recruitment period, 177 patients were diagnosed with EH without atypia on EB. Among which 126 patients were excluded as they did not fulfil the inclusion and exclusion criteria. Among 51 patients enrolled in the study, 25 patients were randomized to group M and 26 to group L (Figure 1). At the end of 6 months, 46 patients were available for histopathological evaluation and 49 patients were available to evaluate the clinical response. Table 1 lists the general information of the study participants. The mean age of the patients was 44 years and the average parity being two. None of the 51 patients were under weight (<18.5 kg/m^2^), 72% of patients in group M and 69% in group L were obese (≥ 25kg/m^2^) (Misra et al., 2009). The pattern of AUB on presentation is depicted in table 1. The patients were distributed equally in both the groups for presenting complaints and general features.

The base line investigations were comparable in both the groups, except for triglycerides (P=0∙019) which was significantly higher in group L. Both the groups were matched for fasting plasma insulin and HOMA- IR as well.

There was negative correlation between BMI and endometrial thickness (ET) in the recruited patients (n=51), but it was not clinically significant (Pearson correlation = -0∙085) (P = 0∙553).But a significant negative correlation between HOMA-IR levels and ET, (Pearson correlation = -0∙297) (P =0∙034) was observed.

*Comparison of treatment response between the groups*

Of the 46 patients who were available for histopathological evaluation after 6 months, 24 of 25 were from group M and 22 of 26 were from group L as depicted in table 2. One patient each from both the groups were not available for post treatment EB. Of the remaining three patients of group L, one patient expelled LNG-IUS in 2 weeks, one patient refused EB at the end of 6 months and one patient underwent hysterectomy in a different centre within 2 months due to persistent bleeding. In group M 100% (24/24) patients showed a complete histopathological response. In group L 95.45% (21/22) patients showed complete response. One patient of group L who did not have a complete response as per outcome assessment had disordered proliferative endometrium on histopathology and was placed in no response category. No patient showed histological evidence of progression of disease. There was no statistically significant (P = 0.47826) difference in primary outcome between the groups.

As elaborated in Table 2, 49 of 51 patients were available for assessment of clinical response through menstrual diary, including those contacted telephonically. Among 49 patients 45 responded clinically with either amenorrhea, regular cycles or spotting. Clinical response was observed in 92% (23/25) patients in group M and 91.67%(22/24) of patients in group L. Among patients who failed to respond, one patient of group M was diagnosed with endometrial polyp at the end of 6 months and underwent hysteroscopic polypectomy which on histopathology was a benign adenomyomatous polyp. There was a significant difference in type of response between the groups at the end of 6 months. Patients on metformin developed amenorrhea (P=0.0053) more frequently while patients in group L developed regular cycles (P=0.027). The mean time of onset of clinical response (reduction in amount of bleeding in subjects with heavy menstrual cycles, and regularization of cycles in subjects with irregular bleeding) in group M was 1∙79 ± 0∙93 months and in group L was 2∙00±1∙38 months, which was not statistically significant (P = 0∙935).

A total six subjects required additional oral progestogens for variable duration for symptomatic relief, one in group M and five in group L. The mean duration of the requirement was 2 months in group M and 3∙20 ±1∙304 months in group L. Two patients of group L underwent hysterectomy during the study period. 

The repeat evaluation of biochemical and haematological parameters at the end of the study showed no significant change in baseline parameters. A decrease in mean weight by 1∙25 ± 3∙096 kg was observed in group M patients, whereas group L patients showed an increase of mean weight by 0.818 ± 3.376 kg, which was statistically significant (P = 0∙0366). The difference in change in BMI between the 2 groups was also significant [P = 0∙023, 95% CI (-1.7802,-0.1418)].There was no significant irreversible adverse effect observed among the subjects during the study period. The observed adverse effects are described in table 2. Following initiation of treatment, subjects in group M had statistically significant nausea. Nausea settled in both the groups in less than two weeks not requiring the termination of treatment. The heaviness in lower abdomen and pain abdomen was managed with analgesics.

**Table 1:** Demographic Characteristics of the Study Subjects, Pattern of AUB at Presentation and Pre-Treatment baseline Investigations of the Subjects

Parameter	Group M n =25	Group L n=26	Total (n=51)	P -value
**Variable**				
(Mean ± Sd)				
Age (years)	44∙2 ± 5∙82	44∙73±5∙96	44∙47±5∙839	0∙749
Parity	2∙24± 0∙93	2∙27±0∙87	2∙25±0∙89	0∙909
Age at menarche (years)	13∙84±1∙57	14∙12±1∙45	13∙98±1∙50	0∙519
BMI (kg/m^2^)	29∙75±6∙85	26∙74±3∙70	28∙22±5∙63	0∙060
**Complaint**, n (%)				
HMB	01 (4∙0%)	05 (19∙2%)	06 (11∙8%)	
IrregMB	07 (28%)	11 (42∙3%)	18 (35∙3%)	
HPMB	14 (56%)	09 (34∙6%)	23 (45∙1%)	0∙131
PMB	03 (12%)	01 (3∙8%)	04 (07∙8%)	
**Investigation** (Mean±SD)				
HAEMOGLOBIN (g/dL)	11∙20 ± 1∙42	11∙12 ± 1∙44		0∙833
T. BILIRUBIN(mg/dL)	0∙61±0∙21	0∙58±0∙25		0∙679
AST (U/L)	27∙88±8.53	26∙26±10∙55		0∙551
ALT (U/L)	29∙27±11∙87	27∙23± 9∙85		0∙508
ALP (U/L)	108∙13±44∙90	116∙68±43∙28		0∙492
UREA (mg/dL)	22∙10±5∙60	23∙97±6∙39		0∙269
CREATININE (mg/dL)	0∙69 ±0∙15	0∙69±0∙20		0∙967
FBS (mg/dL)	90∙35±10∙43	90∙11±7∙78		0∙926
PPBS (mg/dL)	123∙05±22∙45	130∙19±24∙55		0∙283
TRIGLYCERIDE (mg/dL)	107∙91±32∙69	134∙97±45∙76		0∙019
CHOLESTEROL (mg/dL)	187∙74±33∙31	190∙20±38∙84		0∙081
LDL (mg/dL)	121∙96±36∙64	114∙33±33∙82		0∙444
HDL (mg/dL)	46∙19±8∙71	49∙17 ±11∙49		0∙302
FPI (µU/ml)	11∙65±5∙85	9∙73 ±4∙86		0∙210
HOMA- I R (mg/dL)	2∙66±1∙52	2∙15±1∙08		0∙171
ET (mm)	13∙44± 5∙51	13∙90± 5∙18		0∙760

BMI, Body mass index; HMB, Heavy menstrual bleeding; IrregMB, Irregular menstrual bleeding; HPMB, heavy prolonged menstrual bleeding; PMB, postmenopausal bleeding; AST, aspartate transaminase; ALT, Alanine transaminase; ALP, alkaline phosphatase; FBS, fasting blood sugar; PPBS, postprandial blood sugar; LDL, low density lipoprotein; HDL, high density lipoprotein; FPI, fasting plasma insulin; ET, endometrial thickness.

**Table 2:** Histopathological Response to the Treatment, Clinical Response to Treatment, Adverse Effects and Comparison of Change in Parameters after Treatment between the Study Groups

		Group M	Group L	Total		P value
**Histopathological Response**		n= 24^a^ (%)	n = 22^a^(%)	(n =46^a^) (%)		
Complete response	Pill endometrium	17 (70∙83%)	19 (86∙36%)	36 (78∙26%)	45 (97∙83%)	0∙47826
	Late secretory	03 (12∙50%)	02 (09∙09%)	05 (10∙87%)		
	Atrophic	03 (12∙50%)	0 (00∙00%)	03 (06∙52%)		
	Benign polyp	01 (04∙17%)	0 (00∙00%)	01 (02∙17%)		
No response	Disordered proliferative endometrium	00 (00∙00%)	1 (04∙55%)	01 (02∙17%)	1 (02∙17%)	
**Clinical response**		n = 25 (%)	n = 24^b^ (%)	n = 49^b^ (%)		
Amenorrhea		15 (60%)	05 (20∙83%)	20 (40∙82%)		0∙0053
Spotting		03 (12%)	05 (20∙83%)	08 (16∙33%)		0∙463
Regular cycles		05 (20%)	12 (50∙00%)	17 (34∙69%)		0∙027
HMB		02 (08%)	02 (08∙33%)	04 (08∙16%)		1
**Adverse effects** ^c^		n= 25 (%)	n= 26 (%)			
Nausea		10 (41∙67%)	03 (13∙04%)			0∙0265
Heaviness in lower abdomen		05 (20∙83%)	04 (17∙39%)			0∙726
Pain abdomen		05 (20∙83%)	06 (26∙09%)			1
**Investigation**		n=24^a^ (Mean±SD)	n = 22^a^ (Mean±SD)			
HAEMOGLOBIN		0∙83±1∙31	0∙86 ±1∙34			0∙948
T∙ BILIRUBIN		0∙14 ± 0∙20	0∙12 ±0∙45			0∙858
AST		-1∙72 ± 7.42	0∙621 ±8∙69			0∙333
ALT		-1∙14±10∙27	3∙11 ±11∙02			0∙185
ALP		8∙76 ± 35∙54	-4∙18 ±53∙32			0∙344
UREA		0∙71 ± 6∙59	0∙14 ±5∙76			0∙753
CREATININE		-0∙04 ± 0∙17	0∙05 ±0∙15			0∙085
FBS		9∙30 ± 18∙02	6∙17 ±12∙32			0∙492
PPBS		20∙30 ± 33∙38	9∙84 ±32∙18			0∙285
TRIGLYCERIDE		-1∙18 ± 33∙05	-10∙12±38∙90			0∙408
CHOLESTEROL		-7∙27 ±27∙33	-1∙62 ±28∙52			0∙496
LDL		-6∙16 ± 28∙38	10∙67 ± 31∙89			0∙067
HDL		2∙44 ±7∙47	-1∙56 ±7∙48			0∙077
FPI		-1∙06 ±5∙12	-0∙12 ±3∙14			0∙458
BMI		-0∙63±1∙36	0∙34 ± 1∙39			0.023

HMB, Heavy menstrual bleeding; AST, aspartate transaminase; ALT, Alanine transaminase; ALP, alkaline phosphatase; FBS, fasting blood sugar; PPBS, postprandial blood sugar; LDL, low density lipoprotein; HDL, high density lipoprotein; FPI, fasting plasma insulin; BMI, body mass index.^a^, HPE of EB not available for one subject in group A and four in group B; ^b^, Assessment of clinical response was not available in 2 subjects; ^c^, Subjects had more than one complaint (overlap).

## Discussion

Progesterone is the corner stone in the management of EH without atypia. Studies have shown that addition of metformin can enhance the effect of progesterone and also can be as effective as progesterone therapy on its own. The available published literature so far only includes studies comparing use of oral progestogens with metformin for treatment of EH with and without atypia. However, the present study is first of its kind for use of LNG-IUS with metformin in EH without atypia.

The study has also adopted WHO 2014 classification and introduced the use of pictorial menstrual diary for clinical response assessment. A pilot study by Shan et al., (2014) in 16 patients with EH with atypia showed 75% cure rate in group treated with Megestrol Acetate (MA) plus metformin compared to 25% in MA only group during a 3 month follow up. They concluded that metformin plus MA may be a potential alternate therapy for EH with atypia.

In our study, the mean age of subjects, parity and BMI was similar to that observed by Sharifzadeh et al (Sharifzadeh et al., 2017) and Sayyah-melli et al (Sayyah-Melli et al., 2018). The mean HDL levels in the study were less than 50mg/dL suggesting that most of the women met at least one metabolic syndrome criteria. The mean HOMA IR in the study was 2∙402 which being greater than 2 suggests insulin resistance for Indian population (Sinha DP et al., 2009). Even though the mean ET (13.68mm) was comparable to other studies (Sayyah-Melli et al., 2018; Korkmaz et al., 2013), our study did not show any significant correlation between ET and BMI in contrast to prior studies. Earlier studies have demonstrated increase in ET with increased BMI (Douchi et al., 1998; Heller et al., 2011). Our study showed a significant negative correlation between HOMA IR and ET, suggesting early onset of hyperplastic changes in endometrium in individuals with insulin resistance. The European studies have suggested a positive correlation between insulin resistance and ET (Navaratnarajah et al., 2008). Hence more studies are required to know the population-based variations. 

Even though adding metformin to the first line management (LNG-IUS) showed a better histopathological response, it was not statistically significant as noted in the previous studies (Shan et al., 2014; Korkmaz et al., 2013). Seventy eight percent of the total study subjects had pill endometrium on histopathological response assessment and atrophic endometrium was observed only in group M patients (12∙5% ). Tabrizi et al., (2014) observed 87∙5% of atrophic endometrium in metformin group and 66∙7% in MA group. Their study did not have pill endometrium as a pathological entity. Pill endometrium is characterized by inactive endometrial glands in decidualized stroma, which is considered as complete response.

The only subject of the study who did not show a complete histological response was the subject with maximum BMI (34.72 kg/m^2^) in the group L and with a HOMA IR of 3∙5 mg/dl. Women with BMI greater than 34 (37.72, 37.33, 49.73, 41.38kg/m^2^) showed a complete response in group with metformin. There was also a significant decrease in mean BMI in LNG-IUS plus metformin group. We agree with Korkmaz et al that metformin may be used as an adjunctive therapy for persistent EH in women with high BMI (Korkmaz et al., 2013).

In our study, 22.72% subjects of group L required additional oral progesterone compared to only 4.16% in group M to control the symptoms in those who had increased bleeding with LNG-IUS initially. Even though not significant, addition of metformin reduced the increased bleeding associated with LNG-IUS.

Evaluation of clinical response using menstrual diary has not been reported in any other study involving treatment of EH with metformin. In the present study, complete amenorrhea was significantly reported in women treated with LNG-IUS plus metformin. El Behery et al observed 26% amenorrhea in LNG-IUS group compared to none in oral Progestogens group (El Behery et al., 2015).

Only 3.865% subjects from the study underwent hysterectomy for no relief in symptoms, which was in contrast to that observed by Abu et al. where rate of hysterectomy was 22% in individuals treated with LNG-IUS (Abu Hashim et al., 2013).

Like other studies, no severe adverse effects were observed in both arms of the study (Shan et al., 2014). Emily M et al had observed diarrhoea as the most common adverse effect, which was not observed in our study (Emily Meichun Ko et al., 2016).

We observed mean increase in blood sugar levels within both the groups. This finding might have been due to confounding factors like lack of exercise in both the groups, diet, difference in duration of fasting period at the time of blood sampling and also the diet consumed before the postprandial sugar assessment. Estimation of HbA1c at the time of recruitment of subjects into the study would have overcome this disparity and given a meaningful analysis. The present study was not powered to assess a superiority of adding metformin to LNG-IUS in treatment of EH without atypia as a large sample size is required for any conclusive evidence.

Although the present study did not show significant difference in pathological response on addition of metformin, it showed a significant reduction in BMI, lesser need of oral progesterone to control heavy bleeding following LNG-IUS insertion and better bleeding profile. Further study with a larger sample size, targeting obese population is necessary to reveal the additional benefits and to confirm the findings.

## Author Contribution Statement

The authors confirm contribution to the paper as follows: study conception and design: RDR, JK, RS, RB, NS ; data collection: RDR, JK, RB, VJ, VS ; analysis and interpretation of results: RS, RDR, JK; draft manuscript preparation: RDR and JK. All authors reviewed the results and approved the final version of the manuscript.
